# SARS-CoV-2 before and after Omicron: two different viruses and two different diseases?

**DOI:** 10.1186/s12967-023-04095-6

**Published:** 2023-04-10

**Authors:** Renata Gili, Roberto Burioni

**Affiliations:** Università Vita Salute San Raffaele Medical School, via Olgettina 58, 20132 Milan, Italy

**Keywords:** COVID-19, Variant of concern, Omicron, SARS-CoV-2

## Abstract

For the first time in the history of medicine, it has been possible to describe—after a spillover—the evolution of a new human virus spreading in a non-immune population. This allowed not only to observe the subsequent emersion of variants endowed with features providing the virus with an evolutionary advantage, but also the shift of the pathways of virus replication and the acquisition of immunoevasive features. These characteristics had a remarkable influence on the diffusion of the SARS-CoV-2 and on the clinical presentation and prognosis of COVID-19, aspects that are described and commented in this review.

## Background

At the end of 2019 a new coronavirus, the severe acute respiratory syndrome coronavirus 2 (SARS-CoV-2), originated from a spillover and appeared for the first time in a non-immune population, spreading quickly around the world and causing the coronavirus disease 2019 (COVID-19) pandemic, which as of February 2023 caused more than 756 million confirmed cases and over 6.8 million deaths worldwide [1].

Since its emergence, SARS-CoV-2 has given rise to an intense succession of genomic mutations, with the consequent progressive selection of different viral variants. According to the World Health Organization (WHO), a variant of concern (VOC) is a viral variant that has acquired significant enough evolutionary advantages allowing it to prevail over other circulating variants and to have a public health impact [2].

What happened during the COVID-19 pandemic, which had never happened before, is that we were able to observe the evolution of the virus directly and in a very detailed way. This allowed us to evaluate the progressive change of the interaction between the virus and the human host, both from the point of view of the features of the infection and the interaction with the immune system of the host. Indeed, thanks to the molecular tools at our disposal today, viral sequences have been analysed and shared at an unprecedented pace, allowing almost real-time surveillance of the emergence of new mutations potentially linked to changes in viral properties [3, 4], that are the results of a process aimed at optimizing the replicative fitness of the virus and his adaptation to the human host [3].

Furthermore, in a record time during 2020 [5], extremely safe and effective mRNA vaccines against COVID-19 were developed [6, 7]. Thanks to their administration to a remarkable number of individuals in many countries during the early stages of the pandemic we were able, also in this case for the first time in the history of medicine, to see the course of the pandemic changing week by week, due to the progressive increase in the levels of immunity among the population even in absence of infection. From the end of 2019 to today, ideally, we can therefore divide the COVID-19 pandemic that happened in Western countries into two macro-periods: the first in which the population was predominantly non-immune to SARS-CoV-2 and the second when the majority of people acquired a certain degree of immunity, by vaccination or previous infection.

Looking now at the progression of viral variants that we have been able to observe from the beginning of 2020 to today, it seems that during the early stages of the pandemic, in a world of non-immune individuals, the main advantage for SARS-CoV-2 was identified in the acquisition of a higher degree of contagiousness. Almost immediately after its appearance, in fact, SARS-CoV-2 generated the D614G mutation, which quickly become prevalent worldwide, leading to a 20% increase in virus infectivity [8]. Subsequently, the Alpha variant (B.1.1.7) appeared, identified for the first time in the United Kingdom in September 2020 [2] and associated with a rapid growth in confirmed cases of COVID-19 all over the world, recording a further 50% increase in transmissibility if compared to D614G [9]. After that, the Delta variant (B.1.617.2), which first appeared in India in October 2020 [2], has also quickly become globally dominant. Delta had a more marked ability to evade the host immune response than the previous two, in which this property existed but in a very limited way, and combined this feature with a further increase in its transmissibility [10, 11].

On the contrary, the two variants that emerged in parallel to Alfa and Delta and which “focused” mainly on immune evasion rather than on the increase in contagiousness (B.1.351 – Beta and P.1 – Gamma), despite having spread internationally, never became dominant.

The ability to evade the host’s immune response and to reinfect the vaccinated and recovered individuals became, instead, an evolutionary advantage of greater weight only later, when the population immunity grew considerably. Therefore, in a world of non-immune individuals, the increase in transmissibility has proved to be the most important feature characteristic to be acquired from an evolutionary perspective of SARS-CoV-2. On the contrary, immune evasion has become a relevant evolutionary advantage when circulating in a population made mainly of immune individuals, as demonstrated by the emergence of the fifth and last variant defined as VOC by the WHO, the Omicron variant (B.1.1.529), isolated for the first time in South Africa and Botswana in November 2021 and currently prevalent all over the world [12]. With its further increase in contagiousness and its ability to escape the host’s immune response, easily reinfecting vaccinated and recovered individuals, in fact, Omicron has given life to a new phase of the pandemic, breaking the dynamics known up to the moment of its appearance, and has led to an impressive and extremely rapid growth of COVID-19 cases all over the world, overtaking the other circulating variants immediately after its appearance, and spreading with a speed never recorded before.

In this review, we explore the antigenic, virological, clinical, and epidemiological characteristics of the Omicron variant, which demonstrate that the virus has drastically changed and that we are facing a disease in many ways different from the one caused by the pre-Omicron viral variants.

### The antigenic, virological, clinical, and epidemiological characteristics of the Omicron and the pre-Omicron variants

#### Omicron has substantial mutations in the spike protein

The appearance of Omicron immediately aroused global concerns due to its very high number of mutations, strikingly greater than the variants that appeared previously: when compared with the original virus isolated in Wuhan, China, in fact, the Omicron lineages BA.1 and BA.2 (the first to appear) had more than 50 mutations, of which about 30 at the level of the spike protein, the glycoprotein that SARS-CoV-2 uses to enter human cells through binding with its receptor, the angiotensin converting enzyme 2 (ACE2) [13]. These mutations have localized in three fundamental portions of the spike protein. The first is the receptor-binding domain (RBD) – already the main site of many of the mutations that characterized the previous variants [14] –, which binds to the ACE2 and is an important target of many neutralizing antibodies [15]: some of the mutations affecting this site are recognized as responsible for a greater binding affinity to ACE2 [16], as well as for increased resistance to neutralization of the mutated virus by polyclonal and monoclonal antibodies and convalescent plasma [17, 18]. The second portion of the spike protein consists of the amino-terminal (N-terminal) domain (NTD), which is also of crucial importance since many very potent neutralizing antibodies are directed against this site [19, 20]. Finally, the third part endowed with relevant mutations in the Omicron variant is the one located very close to the furin cleavage site, an essential molecular structure for the entry of the SARS-CoV-2 into the host cells (see after); also, these mutations could influence how SARS-CoV-2 enters the host cells [21].

#### Omicron favors a different mode of entry into cells

To enter the host cell, SARS-CoV-2 binds to its obligate receptor, ACE2 [22, 23]. Its entry is allowed first of all by the presence of furin, an enzyme cutting the spike protein of SARS-CoV-2 into two subunits: S1, which binds the ACE2 receptor, and S2, which anchors the spike protein to the cell membrane and mediates membrane fusion. In particular, the link between SARS-CoV-2 and ACE2 induces some changes in the conformation of the S1 subunit, leading to the exposure of a specific part inside the S2 subunit (the S2’ site). At this point, the virus has at least two different routes for entering the cell. In the presence of transmembrane protease serine 2 (TMPRSS2), the S2’ site is cut directly on the surface of the host cell and SARS-CoV-2 enters by fusion (“route 1”). If, on the other hand, the infected cell has insufficient expression of TMPRSS2 or if the virus-ACE2 complex does not encounter TMPRSS2, then SARS-CoV-2 is internalized by endocytosis (“route 2”). At this point, thanks to the acid pH present inside the endosome, the specific enzyme cathepsin becomes active and allows to cut the S2’ site, with the subsequent fusion of the membranes and the release of the virus in the host cell [24].

While pre-Omicron variants preferred the entrance into the host cell by fusion (TMPRSS2 dependent route), Omicron uses more the second route entering the host cell predominantly by endocytosis [25], and this difference can lead to at least two important considerations.

On the one hand, Omicron is less capable of infecting cells of the lower respiratory tract (which have higher levels of TMPRSS2), while prefers to infect cells of the upper respiratory tract, characterized by higher levels of cathepsin, necessary for the entry by endocytosis. In fact, if we compare the replication capacity in the lung cells of the different variants, we note that in these sites Omicron has an attenuated replication, if compared to the others. This correlation suggests that the different entry mode of Omicron may determine preferences in the selection of the tissues that become infected and a different cellular tropism, with a consequent greater infectious capacity at the level of the upper respiratory tract [25, 26].

On the other hand, the Omicron variant is less capable of forming syncytia than the previous variants. The TMPRSS2-mediated membrane fusion, indeed, not only allows entry into the host cell, but also the fusion of the membranes of cells adjacent to each other. The formation of syncytia is associated with a severe clinical picture of COVID-19 and with higher pathogenicity, and while this characteristic was very marked in the Delta variant, it appears to be less present in Omicron [25, 27, 28].

#### Omicron has a greater ability to evade the host's immune response

One of the main characteristics of the Omicron variant is its marked ability to evade the immune response, both in individuals previously infected with SARS-CoV-2 and in vaccinated subjects [29–31]. Furthermore, Omicron BA.4 and BA.5 subvariants can further evade the immune response resulting from previous infections by Omicron BA.1 and BA.2 [32]. This results in a consequent sharp increase in reinfections and breakthrough infections, which are occurring in many countries around the world. However, it is important to note that despite the possibility of reinfection, people who have acquired immunity (whether from vaccination or naturally acquired) are less contagious than non-immune individuals. In particular, the greatest reduction in infectivity occurs in those who have received booster doses and in people who have hybrid immunity, conferred by both vaccination and a previous infection [33]. It is also important to underline that, although Omicron’s own mutations also involve some epitopes recognized by B and T cells, the immune response mediated by these cells remains almost unchanged, and thanks to cellular immunity the symptoms caused by Omicron in vaccinated subjects are, generally, relatively mild [34, 35].

#### mRNA COVID-19 vaccines are less effective against Omicron infections than against previous variants

Unlike what happened with the pre-Omicron variants, against which two doses of mRNA vaccine conferred very strong protection against both infection and severe disease [36], with Omicron two doses of vaccine are close to ineffective and the vaccine protection against SARS-CoV-2 infections after the third dose, although showing an increase, is clearly reduced compared to Delta [25]. In addition, the efficacy of three and four doses of vaccine against infection is further reduced with Omicron subvariants BA.4 and BA.5 compared to the previous ones (BA.1 and BA.2) and fades rapidly. Luckily, the efficacy against severe disease remains preserved at a good level [37]. In particular, this kind of protection is very high when updated bivalent vaccines are used [38].

#### Omicron is more contagious than the previous variants

Despite the high vaccination coverage and high levels of natural immunity achieved worldwide, the Omicron variant has rapidly become globally dominant. From an epidemiological point of view, in fact, it has demonstrated a very high transmission capacity, promptly replacing the Delta variant, dominant in many countries until the end of 2021 [39]. In the United States, for example, the Delta variant counted for 99% of cases on December 4, 2021, but already on January 8, 2022, Omicron caused more than 95% of the total COVID-19 cases [40]. The rapid worldwide growth of the Omicron variant is mainly due to its immune evasive capabilities, responsible for infections of vaccinated or previously infected individuals [41], as well as to its higher tropism for nasal epithelial cells and its higher binding affinity between ACE2 and RBD [40].

The Omicron variant is also continuing to evolve and give rise to several sub-variants. But if Alpha and Delta originated from very distinct branches of the SARS-CoV-2 family tree, all the sub-variants of Omicron originate from a single branch of it and are spreading all over the world, without, at the present, any of them being able to really take over the others, in a phenomenon that has been called “variant swarm” or “variant soup” [42].

#### Omicron causes a different clinical picture than the previous variants

In its evolutionary path, SARS-CoV-2 has progressively reduced the incubation period, which stood at values ​​of around 4.5-5 days for the Alpha, Beta and Delta variants, and dropped to around 3.5 days for the Omicron variant [43]. In addition, clinically, Omicron causes less severe disease than its predecessor variants [44, 45]. Finally, the disease caused by Omicron has different symptoms: the loss of taste and smell is considerably rarer than in the pre-Omicron variants, while the onset of sore throat and hoarse voice is more frequent [46].

### Omicron: a different disease?

Omicron, as we have seen, has distinct characteristics compared to the variants that appeared previously. Its greater transmissibility, the lower efficacy of the vaccines against the infection, the lower severity of the clinical picture with the appearance of different symptoms, the lower incubation period, and the higher replicative efficiency, make this variant a very different virus compared to Alpha, Delta or the strain that contained the D614G mutation.

Going to analyse in more depth the virological, immunological, clinical, and epidemiological characteristics of Omicron, it could, in fact, be hypothesized that SARS-CoV-2 is evolving towards a viral form that causes a more localized infection than the generalized infections caused by pre-Omicron variants. The relationship that a virus establishes with its host from a clinical point of view can, in fact, be summarized with specific patterns.

Generalized viral infections (such as, for example, measles or mumps) are characterized by a long incubation period (about 10–14 days), that follows a viraemia. This long period allows the infected organism to develop a specific immune response, particularly mediated by T lymphocytes. Furthermore, the immunity that the infection (and the vaccine) confers is generally permanent and constituted by a protective IgG antibody response.

On the other hand, the characteristics of localized viral infections (such as, for example, other human coronaviruses or the flu) are very different. In these cases, replication is local, usually at the level of the mucous membranes, the incubation period is shorter, the organism in response to the infection develops a non-specific immune response (mainly mediated by interferon) and protective immunity is mediated by IgA.

Are we sure, therefore, that many Omicron infections that we see localized today in the Delta period wouldn’t present themselves as generalized infections? Several elements described in the evolution of SARS-CoV-2 up to the appearance of Omicron can make us think that this is exactly what is happening. In fact, we are faced with a variant that presents a clear reduction in the incubation period, a lower replicative efficiency in the lung with a lower systemic involvement, parallel to a greater localized replication capacity at the level of the cells of the upper respiratory tract, as well to a vaccine that had very high efficacy in blocking infections with the Alpha and Delta variants, which was lost with the Omicron variant. This last point suggests how, in addition to the ability of Omicron to evade the immune response, maybe comes into play the absence of IgA, necessary for protection at the mucosal level against localized viral infections, since mRNA vaccines currently in use do not stimulate the production of this kind of antibodies.

In conclusion, COVID-19, once the Omicron variant appeared, has somewhat turned into a different disease than the one caused by the previous variants, and for the first time in the history of medicine we have been able to observe and study in a very thorough way the evolution of a new virus spreading through a completely immune-naïve population. However, it is essential to note that even a variant with a lower level of pathogenicity but highly transmissible can continue to pose a significant risk to those most at risk, such as the elderly, those with comorbidities, the immunocompromised patients or those who have not been vaccinated. Furthermore, it should be underlined that our Modern World, with cities of tens of millions of inhabitants and mass air travel, is vulnerable as never before when facing the spread of a contagious infectious disease.

## Data Availability

Not applicable.

## List of abbreviations

SARS-CoV-2: severe acute respiratory syndrome coronavirus 2
COVID-19: coronavirus disease 2019
WHO: World Health Organization
VOC: variant of concern
ACE2: angiotensin converting enzyme 2
RBD: receptor-binding domain
NTD: amino-terminal (N-terminal) domain
TMPRSS2: transmembrane protease serine 2
